# Probiotics and Their Antimicrobial Effect

**DOI:** 10.3390/microorganisms11020528

**Published:** 2023-02-19

**Authors:** Sabina Fijan

**Affiliations:** Faculty of Health Sciences, University of Maribor, Žitna ulica 15, 2000 Maribor, Slovenia; sabina.fijan@um.si

This Special Issue of the journal *Microorganisms* highlights the importance of the antimicrobial effect of probiotics. According to the definition accepted by the World Health Organization and the Food and Agriculture Organization of the United Nations in 2001 and 2002 [1,2] as well as the grammatical update conducted by the Panel of the International Scientific Association for Probiotics and Prebiotics (ISAPP) in 2014 [3], probiotics are defined as “live microorganisms that, when administered in adequate amounts, confer a health benefit on the host.” The antimicrobial or antagonistic activity of probiotics is an important property that includes the production of antimicrobial compounds, such as bacteriocins, competitive exclusion of pathogens, enhancement of the intestinal barrier function in resisting pathogens, as well as enhancing the immune system of the host in order to successfully combat pathogens [4,5]. There are many methods to ascertain antimicrobial probiotic properties, including various in vitro and in vivo methods. The in vitro methods include various modifications of the spot-on lawn assay, agar well diffusion assay (AWDA), co-culturing methods, usage of cell lines and others [6]. The in vivo methods utilize animal models; however, in favour of the protection of animals, alternative methods are being researched to replace all animal research according to the EU directive 2010/63/EU and its consolidated text EUR-Lex—02010L0063-20190626 from 2019 [7,8]. The most important studies on the efficacy of probiotic strains are robust and well-designed, double-blinded randomized placebo-controlled clinical trials that face their own challenges as it is not easy to achieve uniform conditions of participants to eliminate all other influences [6,9]. There is a clear need for more elaborate assays that would better represent the complex interactions between the probiotics and the final host.

The main common probiotics are members of the lactobacilli group, which has recently been divided into 25 genera [10] (including, but not limited to, certain strains of the following species: *Lactobacillus acidophilus, Lactobacillus delbrueckii* subsp. *bulgaricus, Lactobacillus gasseri, Lactobacillus crispatus, Lacticaseibacillus rhamnosus, Lacticaseibacillus casei, Lactiplantibacillus plantarum, Limosilactobacillus reuteri, Levilactobacillus brevis, Ligilactobacillus salivarius* and others), and *Bifidobacterium* genera (e.g., *Bifidobacterium animalis* subsp. *infantis, Bifidobacterium longum, Bifidobacterium bifidum,* and others). Furthermore, certain strains from other bacterial species (e.g., *Lactococcus lactis, Pediococcus mesenteroides, Enterococcus faecium, Streptococcus thermophilus, Bacillus subtilis, Bacillus coagulans, Clostridium butyricum, Escherichia coli*) and even certain strains from certain yeasts (e.g., *Saccharomyces cerevisiae* var. *boulardii*) qualify as probiotics [11]. Lactic acid bacteria constitute a diverse group of Gram-positive, non-spore-forming bacteria, involved in numerous fermentation processes that produce lactic acid from carbohydrates via the homofermentative or heterofermentative pathway [12,13]. The major representatives of this group are *Lactobacillus, Lactococcus, Streptococcus, Leuconostoc, Pediococcus, Enterococcus, Oenococcus* and *Weissella* genera [12]. The *Lactobacillus* genus as well as other lactic acid bacteria have many strains with well-known antimicrobial properties [14]. Cytokine production is also attributed to probiotic lactic acid bacteria, linked to their action in the gut-associated lymphoid tissue that influences host immunity by protecting the host from infections caused by pathogens as well as suppressing allergic symptoms and even cancer [15,16,17]. In the study by Yin and co-authors [15], it was found that the strain *Levilactobacillus brevis* JCM 1059 was most efficient in bacterial uptake by differentiated monocytic THP-1 cells, as well as in subsequent interleukin-12 (IL-12) production. The review by Ahmed and co-authors [17] investigated the antimicrobial and anti-inflammatory effects of various *Weissella* species, and found that they are clinically treatable bacteria with emerging antimicrobial and probiotic benefits ranging from oral health, skin care, obesity, and inflammatory diseases to cancer. 

Current research is focused on finding novel or next-generation probiotic strains with antimicrobial properties that can efficiently modulate the ecological taxa composition and functionality of the human microbiota in the gut and beyond. The most commonly used pathogens to assess the antimicrobial activity in the publication of this issue were from the following genera or species: *Pseudomonas* spp., *Klebsiella* spp., *Escherichia coli*, *Staphylococcus aureus*, *Streptococcus* spp., *Bacillus* spp. and *Salmonella* spp. [18,19,20,21,22]. In the study by Schifano and co-authors [22] several novel *Leuconostoc mesenteroides* strains (C1, C2, C3) and a *Weissella soli* strain (T4), isolated from carrots exhibited strong inhibition against common pathogens. Some strains of the *Bacillus* genus that fulfil the criteria of safety assessment and the status of qualified presumption of safety [23] have also shown an efficient phenotypic antimicrobial effect. Torres-Sánchez and co-authors [18] found that *Bacillus siamenensis*-like strains (rB1, rB3), isolated from the human gut microbiota, were most efficient in antimicrobial activity. Additionally, two potential probiotic strains: *Bacillus subtilis* CP9, isolated from a desert camel, in the study by Sudan and co-authors [20] and *Bacillus subtilis* Fa17.2, isolated from wild *Bromelia* sp. Flowers, in the study by Tenea and co-authors [21], exhibited antimicrobial activity. On the other hand, the strain *Bacillus coagulans* MTCC 5260 used in the study by Fijan and co-authors [19] exhibited only a slight antimicrobial effect against clinical wound pathogens, thus proving the importance of addressing strain specific properties [3,24]. 

Several multi-strain probiotics used in the study by Fijan and co-authors [19], such as OMNi-BiOTiC^®^ dietary supplements, Bio-Kult^®^ and NutriVital Ultra SB, exhibited more efficient antimicrobial action compared to single-strain probiotics, perhaps due to interactions in mixed microbial cultures are driven by metabolite exchanges and are dependent on symbiotic and sometimes competitive behaviours [20]. However, the same study [19] also found that various single strain lactobacilli with well-known antimicrobial properties, including *Lacticaseibacillus rhamnosus* LGG, *Lacticaseibacillus paracasei* Shirota, *Limosilactobacillus reuteri* DSM 17938 and *Lactiplantibacillus plantarum* subsp. *plantarum* DSM 2601 showed efficient antagonistic activity against clinical wound pathogens. The author concluded that perhaps an individualistic approach such as a ‘probiogram’ could be a possibility in the future as a method to find the most efficient targeted probiotic strains, cell-free supernatants, or neutralized cell-free supernatants that have the highest antagonistic effects against individual clinical wound pathogens.

The agar well diffusion assay using cell-free supernatants [18,19,20,21,22] was the most commonly used method to assess the antimicrobial efficiency of the selected probiotics and other beneficial microbes with probiotic properties. Cell-free supernatants are regarded as postbiotics if a beneficial effect to health is observed [25]; thus, efficient antimicrobial effects found in in vitro studies are the first step in establishing new postbiotics. Cell-free supernatants contain metabolites with antimicrobial properties such as bacteriocins, organic acids, including fatty acids, amphiphilic membrane active biosurfactants as well different metabolites with possible antimicrobial effects such as tryptophan-, polyamine-, glutathione- metabolites and others [19,20,21]. Organic acids may have potentiated the activity of other antimicrobial metabolites, which can trigger acidification and/or acid-mediated cell membrane variation to exert an apparent antagonistic effect [21]. Bacteriocins, such as nisins, lactacins, enterocins, colicins, etc., are ribosomal-synthesized peptides or proteins produced by bacterial strains with a strong ability to inhibit pathogenic bacteria and nanoencapsulation prevents proteolytic enzyme degradation and unwanted interactions with food components by enhancing food stability, as found by Shafique and co-authors [26]. Heat stability of antimicrobial substances is also an important trait when selecting bacteriocinogenic producer strains intended to be used as preservation agents in processed foods [21]. The agar spot and co-culturing assays were used in two publications of this issue [19,20]. Both methods investigate the antagonistic effect of viable probiotics against pathogens, where one measures the zone of inhibition of pathogen growth around the spotted probiotic and the other determines the cfu of the pathogen after incubation with the probiotic. 

One route to treat or prevent infectious diseases caused by viruses is the use probiotics. Steyer and co-authors [27] conducted a literature review of randomised placebo controlled clinical studies on the antiviral properties against rotavirus gastroenteric infections in children and Hung and co-authors [28] conducted a literature review on the evidence of oral probiotics as a therapy for the gastrointestinal involvement in COVID-19 patients. Oral probiotics had been evidenced to improve gut health in achieving homeostasis by exhibiting their antiviral effects via the gut–lung axis [29] and patient with COVID-19 have significant changes in fecal microbiomes, characterized by the enrichment of opportunistic pathogens and the depletion of beneficial commensals, which is vastly associated with disease severity [28,30]. Registered clinical trials of probiotics in COVID-19, mainly with lactobacilli and mixtures of bifidobacteria and lactobacilli genera are ongoing and thus the preventive or therapeutic role of probiotics for such patients can be elucidated in the near future [28]. *Saccharomyces cerevisiae* var. *boulardii*, *Lacticaseibacillus rhamnosus* GG, and various multi-strain probiotics exhibited antiviral properties against rotavirus gastroenteric infections in children [27]. The underlying mechanism of the probiotics against rotavirus gastroenteric infections in children included immune enhancement and modulation of intestinal microbiota leading to the shortening of diarrhoea. Many factors influence the outcome of the clinical study, including: correct strain selection and dosage of probiotics, duration of treatment, quality of probiotics as well as the production process of probiotics [27]. More robust, well-designed clinical studies addressing all factors are warranted.

Overall, this Special Issue has brought together new studies on the antimicrobial effects of various novel probiotics from the *Weissella, Bacillus, Leuconostoc* and *Levilactobacillus* genera, as well as well-known probiotic food supplements. It also highlights successful applications of probiotics for different infectious diseases including rotaviral gastrointestinal infections, wound infections and even COVID-19. 
